# Multiple Sublineages of Influenza A Virus (H5N1), Vietnam, 2005−2007

**DOI:** 10.3201/eid1404.071343

**Published:** 2008-04

**Authors:** Tien Dung Nguyen, The Vinh Nguyen, Dhanasekaran Vijaykrishna, Robert G. Webster, Yi Guan, J.S. Malik Peiris, Gavin J.D. Smith

**Affiliations:** * National Institute of Veterinary Research, Hanoi, Vietnam; † University of Hong Kong, Hong Kong Special Administrative Region, People’s Republic of China; ‡ St. Jude Children's Research Hospital, Memphis, Tennessee, USA; § The HKU-Pasteur Research Centre, Pokfulam, Hong Kong Special Administrative Region, China

**Keywords:** highly pathogenic avian influenza, genotype Z, Southeast Asia, virus evolution, molecular epidemiology, dispatch

## Abstract

Phylogenetic analysis of influenza subtype H5N1 viruses isolated from Vietnam during 2005–2007 shows that multiple sublineages are present in Vietnam. Clade 2.3.4 viruses have replaced clade 1 viruses in northern Vietnam, and clade 1 viruses have been detected in southern Vietnam. Reassortment between these 2 sublineages has also occurred.

## Abstract 2

Clade 2.3.4 viruses that are dominant in southern China have now spread to northern Vietnam.

Multiple sublineages of highly pathogenic avian influenza (HPAI) virus (H5N1) have been detected from poultry in Vietnam since 2001 (*1*–*3*). However, the introduction of subtype H5N1 genotype Z virus in 2003 resulted in unprecedented widespread outbreaks in poultry and repeated interspecies transmission to humans; 93 cases were confirmed in Vietnam by the end of November 2005 (*4*,*5*). These viruses became endemic in poultry in Vietnam, causing repeated outbreaks, and have been transmitted to other Southeast Asian countries, where they have caused poultry outbreaks and human infections (*3*–*6*). This virus is designated as clade 1 in the World Health Organization (WHO) influenza (H5N1) virus nomenclature system (*7*). In 2005 a novel reassortant virus of subtype H5N1 (genotype G) from clade 2.3.2 (Mixed/VNM2) was also recognized in Vietnam (*2*). The close phylogenetic relationship of the influenza virus (H5N1) lineages in Vietnam and the southern People’s Republic of China suggests repeated introduction of subtype H5N1 virus into Vietnam (*2*,*3*,*8*). However, the development and evolution of influenza virus (H5N1) in Vietnam since 2005 are not clear.

Since 2003, multiple sublineages of the Goose/Guangdong/1/96 (Gs/GD)–like virus became established in poultry in China (*2*). However, in late 2005, clade 2.3.4 (Fujian-like) influenza virus (H5N1) became dominant and replaced almost all of these previously circulating sublineages; these sublineages have also been been detected in wild birds in Hong Kong Special Administrative Region (SAR), China, and from poultry in Lao People’s Democratic Republic, Malaysia, and Thailand (*3*). Vaccination of poultry in Vietnam against H5 virus was initiated in October 2005. After that, no influenza (H5N1) outbreaks were reported in the country from December 2005 to October 2006 (*9*). However, renewed subtype H5N1 outbreaks in poultry have occurred in Vietnam since November 2006, with evidence of limited human infections during 2007 and 2008. Whether clade 2.3.4 viruses have been introduced into Vietnam is not known.

## The Study

**Figure 1:**
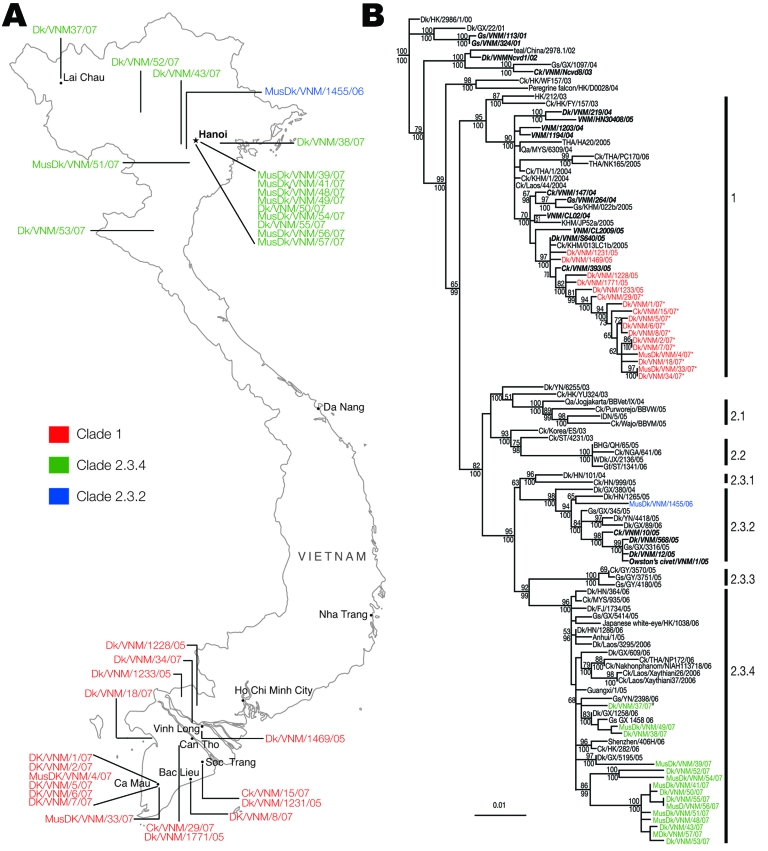
A) Map of Vietnam showing the location of influenza A virus (H5N1) isolation from 2006 to 2007. B) Phylogenetic relationship of the hemagglutinin (HA) gene of influenza A viruses isolated in Vietnam during 2005–2007. Numbers above and below branches indicate neighbor-joining and Bayesian posterior probabilities, respectively. Analyses were based on nt 1–963 of the HA gene. The HA tree was rooted to swan/Hokkaido/51/96. Numbers to the right of the figure refer to World Health Organization influenza (H5N1) clade designations (Table). Virus names described in this study are shown in colored text; previously described Vietnam isolates are shown in ***bold italic*** text. *Denotes viruses with the Ser-123-Pro substitution in the HA. #Denotes a clade 2.3.4 and clade 1 reassortant virus. Scale bar, 0.01 substitutions per site. BHG, bar-headed goose; Ck, chicken; Dk, duck; FJ, Fujian; Gs, goose; GX, Guangxi; GY, Guiyang; HK, Hong Kong Special Administrative Region, People’s Republic of China; HN, Hunan; JX, Jiangxi; IDN, Indonesia; KHM, Cambodia; MusDk, muscovy duck; MYS, Malaysia; NGA, Nigeria; Qa, Quail; ST, Shantou; THA, Thailand; VNM, Vietnam; WDk, wild duck; YN, Yunnan.

We sequenced the whole genomes of 33 avian influenza virus (H5N1) isolates collected during poultry outbreaks in Vietnam from October 2005 through May 2007. All sequences that were generated in this study have been deposited in GenBank (CY029508–CY029771). The virus was primarily detected in aquatic poultry (ducks, muskovy ducks), but it was also isolated from 2 chickens in December 2006 and January 2007. The date and location of virus isolation are summarized in the Table and Figure 1, **panel A**.

**Table:** Influenza virus isolates from poultry in Vietnam, 2005–2007

Isolate*	Genotype	Date	Province†	Sublineage‡
Dk/VNM/1228/05	Z	2005 Oct	Dong Thap (S)	Clade 1
Dk/VNM/1231/05	Z	2005 Nov	Soc Trang (S)	Clade 1
Dk/VNM/1233/05	Z	2005 Nov	An Giang (S)	Clade 1
MusDk/VNM/1455/06	G	2006 Feb	Ha Tay (N)	Clade 2.3.2
Dk/VNM/1469/05	Z	2005 Nov	Vinh Long (S)	Clade 1
Dk/VNM/1771/05	Z	2005 Oct	Can Tho (S)	Clade 1
Dk/VNM/1/07	Z	2007 Jan	Ca Mau (S)	Clade 1
Dk/VNM/2/07	Z	2007 Jan	Ca Mau (S)	Clade 1
MusDk/VNM/4/07	Z	2007 Jan	Ca Mau (S)	Clade 1
Dk/VNM/5/07	Z	2007 Jan	Ca Mau (S)	Clade 1
Dk/VNM/6/07	Z	2007 Jan	Ca Mau (S)	Clade 1
Dk/VNM/7/07	Z	2007 Jan	Ca Mau (S)	Clade 1
Dk/VNM/8/07	Z	2007 Jan	Bac Lieu (S)	Clade 1
Ck/VNM/15/07	Z	2007 Jan	Soc Trang (S)	Clade 1
Dk/VNM/18/07	Z	2007 Jan	Kien Giang (S)	Clade 1
Ck/VNM/29/07	Z	2006 Dec	Hau Giang (S)	Clade 1
MusDk/VNM/33/07	Z	2007 Jan	Ca Mau (S)	Clade 1
Dk/VNM/34/07	Z	2007 Jan	Can Tho (S)	Clade 1
Dk/VNM/37/07	Z	2007 Mar	Lai Chau (N)	Clade 2.3.4
Dk/VNM/38/07	Z	2007 Mar	Hai Duong (N)	Clade 2.3.4
MusDk/VNM/39/07	Z	2007 Mar	Hanoi (N)	Clade 2.3.4
MusDk/VNM/41/07	Z	2007 Mar	Hanoi (N)	Clade 2.3.4
Dk/VNM/43/07	Z	2007 Apr	Ha Tay (N)	Clade 2.3.4
MusDk/VNM/48/07	Z	2007 May	Hanoi (N)	Clade 2.3.4
MusDk/VNM/49/07	Z	2007 Apr	Hanoi (N)	Clade 2.3.4
Dk/VNM/50/07	Z	2007 May	Hanoi (N)	Clade 2.3.4
MusDk/VNM/51/07	Z	2007 Apr	Ha Nam (N)	Clade 2.3.4
Dk/VNM/52/07	Z	2007 May	Phu Tho (N)	Clade 2.3.4
Dk/VNM/53/07	Z	2007 May	Nghe An (N)	Clade 2.3.4
MusDk/VNM/54/07	Z	2007 May	Hanoi (N)	Clade 2.3.4
Dk/VNM/55/07	Z	2007 May	Hanoi (N)	Clade 2.3.4
MusDk/VNM/56/07	Z	2007 May	Hanoi (N)	Clade 2.3.4
MusDk/VNM/57/07	Z	2007 Apr	Hanoi (N)	Clade 2.3.4

*Ck, chicken; Dk, duck; MusDk, muscovy duck; VNM, Vietnam. †The letters S and N denote southern Vietnam and northern Vietnam, respectively. ‡Based on the World Health Organization influenza (H5N1) nomenclature system (*7*).

To understand the developments of influenza virus (H5N1) in Vietnam, we characterized all 8 gene segments of these 33 viral isolates and phylogenetically analyzed them with all available influenza virus (H5N1) previously isolated from Vietnam, Thailand, Malaysia, Lao People’s Democratic Republic, and southern China and with reference viruses belonging to each of the designated clades of the WHO influenza (H5N1) nomenclature system. Sequence assembly, editing, multiple sequence alignment, neighbor-joining, and Bayesian phylogenetic analyses were conducted as previously described (*3*). Maximum-likelihood trees were constructed by using Garli version 9.04 (*10*).

The hemagglutinin (HA) genes of all 33 Vietnam isolates were derived from the Gs/GD-like lineage; however, they fell into 3 distinct sublineages (Figure 1, **panel B**). Seventeen of 33 isolates analyzed were clade 1; however, 15 isolates between March and May 2007 belonged to clade 2.3.4. A single virus isolated in February 2006 (Muscovy duck/Vietnam/1455/2006) clustered within clade 2.3.2.

Phylogenetic analyses also showed a geographic distinction among the isolates characterized in this study. Isolates from samples taken in the northern provinces of Vietnam belonged to clades 2.3.2 and 2.3.4, whereas all isolates in the southern provinces of Vietnam were clade 1 (Figure 1). The clade 1 viruses isolated in the southern provinces from October 2005 through January 2007 were most closely related to viral isolates from poultry in Cambodia in the same period. When one considers the shared land border between Cambodia and southern Vietnam, the close relatedness of these viruses is reasonable. Two of the human influenza (H5N1) cases that were detected from Cambodia were located near the Vietnam-Cambodia border region (*11*). The genetic similarity of strains of influenza virus (H5N1) in Cambodia and southern Vietnam have been observed since 2004 (*4*).

The clade 2.3.2 and clade 2.3.4 viruses isolated from northern Vietnam were most closely related to virus isolated from poultry in Guangxi, China (Figure 1, **panel B**). Because Guangxi shares a border with Vietnam and trade is extensive between those regions, these viruses were most likely introduced into northern Vietnam through poultry trade. However, the clade 2.3.4 viruses from Vietnam do not form a monophyletic group within clade 2.3.4. Therefore, unlike the single introduction of clade 1 viruses in 2003 (*4*), these results raise the possibility of multiple introductions of the clade 2.3.4 viruses into northern Vietnam.

**Figure 2:**
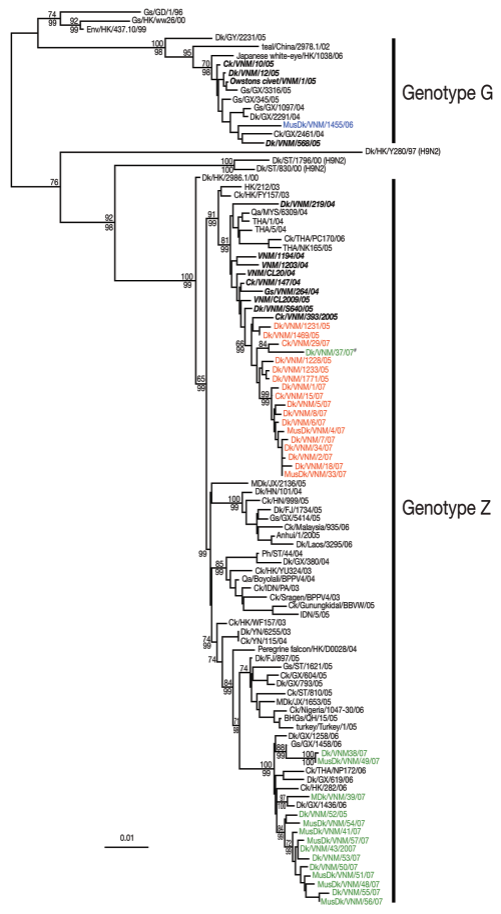
Phylogenetic relationship of the polymerase basic 2 (PB2) gene of influenza A viruses isolated in Vietnam from 2006 to 2007. Numbers above and below branches indicate neighbor-joining and Bayesian posterior probabilities, respectively. Analyses were based on nt 986–2,288 of the PB2 gene. The tree was rooted to equine/Prague/1/56. Viruses names described in this study are shown in colored text; previously described Vietnam isolates are shown in ***bold italic*** text. #Denotes a clade 2.3.4 and clade 1 reassortant virus. Scale bar, 0.01 substitutions per site. BHG, bar-headed goose; Ck, chicken; Dk, duck; FJ, Fujian; Gs, goose; GX, Guangxi; GY, Guiyang; HK, Hong Kong Special Administrative Region, People’s Republic of China; HN, Hunan; JX, Jiangxi; IDN, Indonesia; KHM, Cambodia; MusDk, muscovy duck; MYS, Malaysia; NGA, Nigeria; Qa, Quail; ST, Shantou; THA, Thailand; VNM, Vietnam; WDk, wild duck; YN, Yunnan.

Phylogenetic analyses of the neuraminidase gene and all internal gene segments (data not shown) show that while most of the isolates were genotype Z viruses, they also formed distinct groups that were broadly similar to the evolutionary relationships seen in the HA tree. Duck/Vietnam/37/2007, which belongs to clade 2.3.4, shared the internal gene constellation of clade 1 viruses (Figure 2), providing evidence for cocirculation of these virus groups and evidence of reassortment between different sublineages within Vietnam influenza (H5N1) isolates. A single influenza (H5N1) genotype G virus (Muscovy duck/Vietnam/1455/2006) was also identified in February 2006, which indicates that genotype G viruses may be persistent in poultry in Vietnam (Figure 2). These results confirm that the genetic diversity of strains of influenza virus (H5N1) in Vietnam is similar to that in southern China during the same period (*3*).

The motif of multiple basic amino acids at the HA cleavage site that is characteristic of HPAI was maintained in all viruses characterized; however, clade-specific mutations were observed in different sublineages, consistent with previous reports (*3*,*4*,*8*,*12*). The receptor-binding pocket of HA1 retains Gln 222 and Gly 224 (H5 numbering), which preferentially binds avian-like α2,3-NeuAcGal linkages. However, all 12 clade 2.3.4 viruses and the single clade 2.3.2 virus have an Arg-212-Lys mutation in the HA, whereas 12 clade 1 viruses (marked on Figure 1) have a Ser-123-Pro mutation, previously reported from a Vietnam influenza (H5N1) human isolate (*8*), which has been associated with receptor binding. The importance of this change is not clear (*13*,*14*).

Mutations in the Matrix protein 2 ion channel associated with amantadine resistance were detected in all clade 1 virus isolates tested. These viral strains retained the dual mutations Leu26Ile and Ser30Asn in the M2 protein similar to previous clade 1 strains (*15*). No mutation associated with amantadine resistance was recognized in those clade 2.3.2 and clade 2.3.4 strains except the reassortant Dk/VNM/37/07, which had an additional Val-27-Ala mutation in the M2 protein. Thus we recorded an HPAI (H5N1) virus strain with a triple mutation associated with amantadine resistance. All viruses characterized do not have mutations in the NA gene that confer resistance to oseltamivir. Other known virulence mutations, including at polymerase basic protein 2 position 627, were not present in any of the viruses characterized.

## Conclusions

This study confirms that clade 2.3.4 virus sublineages that are dominant in southern China have now spread to northern Vietnam (*3*). These viruses appear to have replaced the clade 1 viruses in northern Vietnam just as previous influenza (H5N1) sublineages were replaced in southern China (*3*); however, clade 1 viruses are still detected in the southern provinces of Vietnam. It is, therefore, possible that the clade 1 viruses in southern Vietnam may eventually be replaced by clade 2.3.4. The availability of extensive genetic data from southern China enables us to recognize the development of influenza virus (H5N1) in Vietnam and indicates that clade 2.3.4 viruses may have been introduced into Vietnam on multiple occasions. However, because systematic surveillance data are lacking, determining the interaction of viruses between the northern and southern provinces of Vietnam, and also between different countries in Southeast Asia, is not possible. When one considers that multiple sublineages of influenza virus (H5N1) are simultaneously endemic to Southeast Asia, systematic surveillance in poultry remains essential to understand the further evolution of this subtype in this region and the potential for pandemic emergence, as well as to monitor the efficacy and cross-protection of poultry vaccines.
